# Organizational contextual factors that predict success of a quality improvement collaborative approach to enhance integrated HIV-tuberculosis services: a sub-study of the Scaling up TB/HIV Integration trial

**DOI:** 10.1186/s13012-021-01155-7

**Published:** 2021-09-17

**Authors:** Santhanalakshmi Gengiah, Catherine Connolly, Nonhlanhla Yende-Zuma, Pierre M. Barker, Andrew J. Nunn, Nesri Padayatchi, Myra Taylor, Marian Loveday, Kogieleum Naidoo

**Affiliations:** 1 Centre for the AIDS Programme of Research in South Africa (CAPRISA), Nelson R Mandela School of Medicine, University of KwaZulu-Natal, Private Bag X7 Congella, Durban, 4013 South Africa; 2 School of Nursing and Public Health, University of KwaZulu-Natal, Durban, South Africa; 3 CAPRISA-MRC TB-HIV Pathogenesis and Treatment Research Unit, Durban, South Africa; 4 Institute for Healthcare Improvement, Cambridge, MA USA; 5 Gillings School of Global Public Health, University of North Carolina (UNC),Chapel Hill, Chapel Hill, USA; 6 Medical Research Council, Clinical Trials Unit at University College London (UCL), London, UK; 7 HIV Prevention Research Unit, South African Medical Research Council, Durban, South Africa

**Keywords:** Quality improvement collaboratives, HIV-TB integration, Cluster-randomized trial, Organizational contextual factors, South Africa

## Abstract

**Background:**

A quality improvement (QI) collaborative approach to enhancing integrated HIV-Tuberculosis (TB) services may be effective in scaling up and improving the quality of service delivery. Little is known of the role of organizational contextual factors (OCFs) in influencing the success of QI collaboratives. This study aims to determine which OCFs were associated with improvement in a QI collaborative intervention to enhance integrated HIV-TB services delivery.

**Methods:**

This is a nested sub-study embedded in a cluster-randomized controlled trial. Sixteen nurse supervisors (clusters) overseeing 40 clinics were randomized (1:1) to receive QI training and mentorship, or standard of care support (SOC). In the QI arm, eight nurse supervisors and 20 clinics formed a “collaborative” which aimed to improve HIV-TB process indicators, namely HIV testing, TB screening, isoniazid preventive therapy (IPT) initiations, viral load testing, and antiretroviral therapy for TB patients. OCFs measured at baseline were physical infrastructure, key staff, flexibility of clinic hours, monitoring data for improvement (MDI), and leadership support. Surveys were administered to clinic staff at baseline and month 12 to assess perceptions of supportiveness of contexts for change, and clinic organization for delivering integrated HIV-TB services. Linear mixed modelling was used to test for associations between OCFs and HIV-TB process indicators.

**Results:**

A total of 209 clinic staff participated in the study; 97 (46.4%) and 112 (53.6%) from QI and SOC arms, respectively. There were no differences between the QI and SOC arms scores achieved for physical infrastructure (78.9% vs 64.7%; *p* = 0.058), key staff (95.8 vs 92; *p* = 0.270), clinic hours (66.9 vs 65.5; *p* = 0.900), MDI (63.3 vs 65; *p* = 0.875, leadership support (46.0 vs 57.4; *p* = 0.265), and perceptions of supportiveness of contexts for change (76.2 vs 79.7; *p* = 0.128 and clinic organization for delivering integrated HIV-TB services (74.1 vs 80.1; *p* = 0.916). IPT initiation was the only indicator that was significantly improved in the parent study. MDI was a significantly associated with increasing IPT initiation rates [beta coefficient (*β*) = 0.004; *p* = 0.004].

**Discussion:**

MDI is a practice that should be fostered in public health facilities to increase the likelihood of success of future QI collaboratives to improve HIV-TB service delivery.

**Trial registration:**

Clinicaltrials.gov, NCT02654613. Registered 01 June 2015.

**Supplementary Information:**

The online version contains supplementary material available at 10.1186/s13012-021-01155-7.

Contributions to the literature

- QI uptake maybe enhanced in settings where monitoring data for improvement has been a routine practice.
- The effects of QI interventions are enhanced in contexts that are supportive of change and well organized for delivering integrated HIV-TB services.
- The Context Assessment for Community Health tool should be considered for rapid assessment of whether a setting is receptive and ready for change.
- Fostering a culture of using data for improvement can be facilitated by ensuring data is accurate and accessible to clinic teams.

## Background

Among high burden countries for tuberculosis (TB), South Africa ranks second highest for TB incidence rates, estimated at 615 cases per 100 000 population [1]. Fifty-eight percent of new TB cases are co-infected with HIV and mortality rates among HIV-TB co-infected cases (62 per 100 000 population) are double that of TB mono-infected cases (38 per 100 000 population) [1]. The World Health Organization’s End TB Strategy set ambitious targets to reduce TB incidence and mortality by 90% and 95%, respectively, by 2035 [2]. South Africa has a significant contribution to make in achieving these targets and addressing the HIV-TB burden is a key public health priority [3]. To this end, the South African National Department of Health treatment guidelines, recommend integrated HIV-TB services, care, and treatment as routine care [4]. Recent studies have highlighted gaps in integrated HIV-TB service delivery such as patients missed for screening and diagnosis of HIV and TB [5–7]; missed viral load monitoring [8]; and sub-optimal coverage of TB prevention treatment for eligible HIV patients [1].

Missed opportunities to offer HIV-TB services to patients already accessing healthcare point to health systems weaknesses at the frontline of healthcare. Quality improvement (QI) methods offer an ideal solution to improve underlying systems for HIV-TB service delivery [9]. QI collaboratives offer a potentially effective strategy to facilitate scale-up of best practices in HIV-TB service delivery [9]. While there are many adaptions of QI collaboratives, the essential components include (i) different facility teams work together to improve performance on a common health topic, led by a faculty of experts; (ii) sharing of experiences, change ideas, and best practices between clinic teams; and (iii) mentorship of clinic teams to develop and rapidly test change ideas for a given improvement aim [10]. This approach is premised on the principle that group learning accelerates the generation of change ideas and optimally utilizes experts to facilitate learning and inform best practices [10, 11].

First becoming popular in high-income countries before spreading to low- and middle-income countries, QI collaboratives are widely adopted and utilized for improvement in a multitude of health topics since their introduction over 30 years ago [10, 11]. As the strategy proliferated, concerns regarding lack of clear evidence of effectiveness, cost-effectiveness, replicability, and sustainability have been raised [10–15].

A systematic review of QI to improve antiretroviral (ART) uptake reported modest improvement with wide variations between QI collaboratives from one setting to the next; median improvement was 22% ranging from 12.8 to 29.8% [16]. Similarly, a review of 29 QI collaboratives, specifically from low- and middle-income countries, found variations in improvement; however, larger improvements were more likely when a training component was added to the QI collaborative strategy as opposed to QI collaborative alone [14]. On its own, QI collaboratives showed no to little improvement in patients’ outcomes (median effect size (MES) less than 2%); however, combined with a training component, both patients’ outcomes (MES of 111.6%) and healthcare provider practice outcomes (MES from 52.4–63.4%) improved [14].

The variation between settings suggests that what works in one setting may not work in other settings [10]. Much of the explanations for the variations is attributed to “organizational context” and the inherent differences and uniqueness of organizations, individuals, and teams from one setting to another [17]. The Promoting Action on Research Implementation in Health Services (PARIHS) framework defines “context” as the environment or setting in which people receive healthcare services, or the environment in which the proposed change is to be implemented [18, 19].

The few studies that investigated contextual factors influencing the QI outcomes, attribute variations to baseline performance (low performing indicators have a larger room for improvement) [13], simplicity of interventions [20], and clinic team characteristics such as leadership, access to resources, and clinical skills [21, 22]. In recent literature, supportiveness of organizational contexts for change is emerging as a key factor for implementing new interventions or changes [23, 24]. Given the use of experts, time away from clinics to attend collaborative meetings, and in-person mentorship activities, QI collaboratives represent a substantial investment in time and resources and have been cited as costly [12]. Understanding which and how contextual factors impact QI collaboratives is important to enhance success and sustainability of this strategy [11, 17].

The Scaling up TB/HIV Integration (SUTHI) trial tested the effectiveness of a QI collaborative approach to enhancing integrated HIV-TB services [9]. This is a sub-study of the SUTHI trial, to determine which organizational contextual factors influenced the QI intervention to improve HIV-TB services so that these factors can be strengthened in future scale-up efforts. A secondary objective was to determine if there were any major differences in organizational contextual factors (OCF) in the QI arm compared to the standard of care arm (comparator group) which may explain the differences in HIV-TB process outcomes observed in the two study arms.

## Methods

### Study design: The Scaling Up TB HIV trial

The design and rationale for the SUTHI trial are published elsewhere [9]. Briefly, SUTHI was a cluster-randomized trial to determine the effectiveness of QI methods in integrating HIV-TB services on mortality in TB, HIV, and HIV-TB patients [9]. Sixteen nurse supervisors (clusters) and the 40 primary healthcare (PHC) clinics under their oversight, were randomly assigned (1:1) to either a QI intervention (hereafter known as the QI arm) or to standard of care (SOC) support and supervision (hereafter known as the SOC arm). Eight nurse supervisors and their 20 clinics were assigned to the QI arm and eight nurse supervisors and their 20 clinics were assigned to the SOC arm. The study was implemented in the Ugu and King Cetshwayo Districts of KwaZulu-Natal, South Africa from 01 December 2016–31 December 2018. All study clinics were followed up for 18 months.

### Study design: Organizational contextual factors nested sub-study

This is a nested sub-study of the SUTHI trial which was designed to collect data on OCFs that may influence improvement of integrated HIV-TB service delivery and explain why the QI intervention was successful or not. Parallel to the implementation of the parent study, OCFs were assessed at set study time points using surveys administered to consenting clinic staff, and study exit focus group discussions (FGDs) conducted with clinic staff from both study arms.

### The intervention: The Breakthrough Series Collaborative

**Fig. 1:**
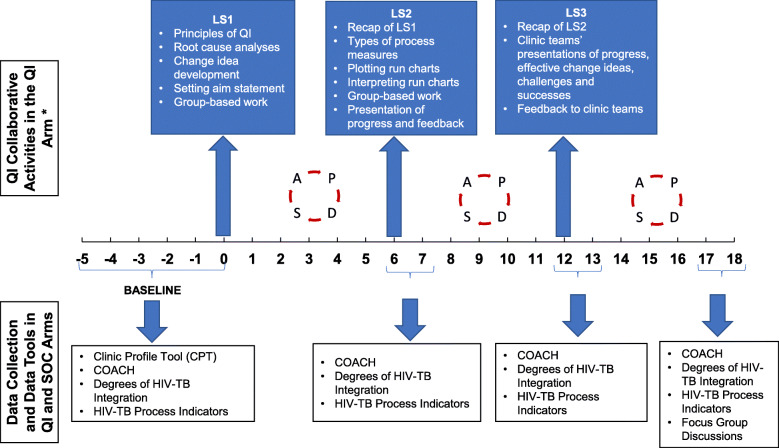
Timing of QI activities and data collection in the SUTHI trial. COACH, Context Assessment in Community Health; PDSA, Plan-Do-Study-Act; LS, learning session; QI, quality improvement; SOC, standard of care; TB, tuberculosis. *The standard of care arm received standard support and supervision for HIV-TB integration

The SUTHI trial adopted a QI intervention structured as a Breakthrough Series Collaborative [25]. Nurse supervisors and clinics in the QI arm formed the QI collaborative. The collaborative met for three 2-day learning sessions timed at 6-month intervals. Learning sessions included coursework on the principles and practice of QI methods and interactive group-based work. Figure 1 illustrates the topics covered at each learning session. Six-month intervals allowed clinic teams time to develop and test changes ideas, and acquire best practices to present to each other. Between learning sessions, a QI nurse mentor, made in-person visits to clinics and provided QI mentorship, reinforced knowledge from learning sessions, and reviewed clinic data. The Model for Improvement was the methodological framework to identify, develop and test change ideas [26]. Rapid, plan-do-study-act cycles facilitated the development and testing of change ideas at the clinic level. QI mentorship visits were fortnightly for the first 12 months and reduced to once a month for the last six months of the study period.

The QI collaborative worked toward a single goal of improving integrated HIV-TB service delivery and focused on eight HIV-TB process indicators, namely: HIV Testing Services (including testing TB patients); TB screening; isoniazid preventative therapy (IPT) for eligible HIV patients; ART for all HIV-TB patients; cotrimoxazole therapy for HIV-TB co-infected patients; retention in care strategies; enhanced treatment adherence strategies including, viral load testing coverage; and a single integrated data management system for both HIV and TB data.

### Identification of organizational contextual factors

The PARIHS framework contributed to defining and identifying key OCFs measured in this sub-study [19]. The framework proposes that successful implementation of evidence is a function of three inter-related key elements: (i) the strength of the evidence being implemented, (ii) the supportiveness of the context in which implementation is occurring, and (iii) the facilitation mechanism used to introduce change [19]. In this paper, reference to ‘organizational context’ pertains to the clinic-level where care is provided, and OCFs are the elements of organizational context that facilitate the adoption of changes.

The PARIHS framework identified key elements of a supportive organizational context, namely: physical infrastructure, human resources, leadership support, monitoring and evaluation of performance, and receptiveness of contexts to implement changes [19]. These key elements were adopted and assessed in this sub-study. In addition, we reviewed other studies that measured clinic-level factors and identified flexibility in clinic hours, and clinic-level organization and planning for integrated HIV-TB service delivery, as elements of organizational context that were relevant to this sub-study [27, 28]. In Table 1, we define each the OCFs assessed in this study.

**Table 1:** Definition and measurement of organizational contextual factors

Organizational contextual factors (OCFs)	Definition	Allocation of scores	Max score per clinic	Method	Completed by	Survey used
Physical Infrastructure	Refers to availability, utilization, and cleanliness of spaces, rooms, and facilities that are required for patient care, consultation rooms, waiting areas, designated cough booth, designated pharmacy, privacy for patients, vitals assessment* room, and ablution facilities.	1 point allocated to each area for each attribute of availability, utilization, and cleanliness Availability = 7 Utilization = 7 Cleanliness = 7	21	Key areas were directly observed and scored.	Jointly completed by study staff and facility manager or designee	Physical infrastructure is a sub-scale located in the CPT
Key staff	Refers to frontline healthcare workers that are considered key personnel in providing patient care and monitoring delivery of healthcare services at the clinic level. Key staff included: - Facility manager - NIMART nurse - PN trained to initiate and manage TB treatment - Lay counsellors - Data capturer - Enrolled nurses	1 point allocated if key staff post was filled at the time of completing the survey	6	Data received directly from facility manager or designee	Jointly completed by study staff and facility manager or designee	Key staff is a sub-scale located in the CPT
Flexibility of clinic hours	Refers to the operating hours of clinics as a proxy measure for the extent to which clinic services are available to the community. Normal hours were defined as Monday to Friday from 07:00 to 16:00. Flexibility is defined as normal hours plus any hours on either side of normal hours or normal hours plus weekends or public holidays	Availability of clinic services during normal working hours = 1 point; extended hours = 2 points; weekends, extended hours, and public holiday = 3 points	3	Data received directly from facility manager or designee	Jointly completed by study staff and facility manager or designee	Flexibility of clinic hours is a sub-scale located in the CPT
Leadership support *	Refers to leadership support visits from the DMT conducted within the last 6 months. Key DMT staff considered were: TB manager, HAST manager, QA manager, M&E manager. Frequency with which the facility manager** was off-site for meetings was considered and combined with the leadership visits score.	1 point allocated to each of the 4 DMT members who visited the clinic even once in the last 6 months **plus** Frequency facility manager is off-site: Weekly = 1 Bi-monthly = 2 Monthly = 3 Quarterly = 4	8	Data received directly from facility manager or designee and confirmed with the Clinic Visitor’s logbook	Jointly completed by study staff and facility manager or designee	Leadership support is a sub-scale of the CPT
Monitoring data for improvement (MDI)	Refers to the extent to which clinic teams have accessed and utilized integrated HIV and TB electronic databases, met to discuss performance, and monitors HIV and TB programme outcomes.	Key systems in place for MDI allocated 1 point each and evidence of implementation allocated 1 point each: - Team information meetings—2 - -Ability to generate reports from the patient electronic database—2 - HIV-TB mortality data reviewed—2 - Single electronic system for HIV and TB—2 - Data quality assurance systems in place and implemented—2 - Clinic improvement team available and functional—2	12	Data received directly from facility manager or designee Team meetings verified by meeting minutes. Direct observation of integrated electronic and patient file system Data quality assurance plans observed on file	Jointly completed by study staff and facility manager or designee	Monitoring data for improvement is a sub-scale located in the CPT
Supportive contexts for change	Refers to clinic staff perceptions of the extent to which their work environment was supportive to making changes.	The COACH survey scored as per developers’ guidance which was to calculate the mean of all sub-scale means	Mean of 5	Survey administered to clinic staff volunteers by a trained study staff member	Clinic staff who volunteered and agreed to sign the informed consent	COACH tool
The degree of integrated TB and HIV services	Validated survey that assessed the perceptions of healthcare workers in the extent to which staff and clinic processes were organized and coordinated toward integrated HIV-TB services	Degree of integrated TB and HIV survey as per developer’s guidance which was to calculate the mean of all sub-scale means	Mean of 5	Survey administered to clinic staff volunteers by a trained study staff member	Clinic staff who volunteered and agreed to sign the informed consent	Degree of integrated TB and HIV survey

*CPT* Clinic Profile Tool, *DMT* District Management Team, *HAST* HIV/AIDS/STI and TB, *M&E* monitoring and evaluation, *COACH* Context Assessment for Community Health, *NIMART* Nurse-Initiated Management of Antiretroviral Therapy, *OCF* organizational contextual factors, *PN* professional nurse, *QA* quality assurance, *TB* tuberculosis

*The scoring of the Leadership sub-scale deviated from the original plan to give regular visits higher scores. We learnt that DMTs are mandated to visit clinics quarterly. Quarterly scores would have been assigned a score of 1 which would have been an inaccurate reflection of the leadership support. Instead, we rephrased the question, to capture if any leadership visits had occurred in the last 6 months from the time the questionnaire was administered

**On-site leadership support is often compromised by the demand placed on facility managers to attend meetings hence we included this item in the leadership support sub-scale

### Data collection tools and surveys

We searched for piloted, validated, and published measures to quantitatively assess the selected OCFs. We adopted tools appropriate for low-and middle-income countries and where no tool was available or appropriate, we designed a tool in-house. In this sub-study, three surveys were used, the Clinic Profile Tool (CPT), The Context Assessment for Community Health (COACH) survey, and the Degrees of integrated Tuberculosis and HIV services survey. Figure 1 illustrates the study time points at which each survey was administered and Table 1 shows who were involved in completing the surveys.

#### The Clinic Profile Tool

The Institute for Healthcare Improvement (IHI) provided a survey, routinely used in past QI projects, to assess resources at facilities and we amended the survey in collaboration with an IHI QI advisor. Amendments included using words and terms that were familiar to clinic staff in our setting and we added on items pertaining to integration of HIV and TB systems. The CPT contained several sub-scales; however, we only assessed the following: physical infrastructure, key staff availability, flexibility of clinic hours, monitoring data for improvement, and leadership support from the District Health Offices. This survey was completed jointly by a trained study staff member and the clinic facility manager and in some instances direct observation by study staff were used to confirm responses. All responses were binary, that is, either a “yes” or “no” was required. Table 1 shows the scoring method used to assess each OCF. The CPT was administered at baseline only (Fig. 1). Due to limited study resources and time, the CPT was not validated. Additional file 1 contains the full CPT.

#### Supportiveness of contexts for change

To assess clinic staffs’ perceptions of the supportiveness of contexts to implement changes, we used a validated survey, called the Context Assessment for Community Health (COACH) survey. Developed by Bergstrom et al. (2015), the COACH was designed to measure the extent to which nurses, physicians, midwives, and community health perceived their work environment as receptive and prepared for implementing changes [23]. We extended the administration of the COACH survey to non-clinically trained staff. The survey has eight sub-scales, namely: resources, community engagement, monitoring services for action, knowledge sources, commitment to work, work culture, leadership, and informal payment (Additional file 2). Sub-scale items are phrased as statements to which respondents could agree or disagree on a 5-point Likert-type scale;1 =Strongly Disagree and 5=Strongly Agree. The COACH survey had a Cronbach’s Alpha score of > 0.70, which is an indication that items similar to each other are highly correlated and this is reflective of a reliable tool [23] The COACH survey was administered at baseline and months 6, 12 and 18 of the study (Fig. 1).

Importantly, some sub-scales in the COACH survey overlap with the CPT (Leadership, Resources, and Monitoring data for improvement); however, the defining characteristic is that the COACH measures perceptions of clinic staff and the CPT was a relatively more objective measure where direct observation and verification of data were used.

#### Degree of integrated tuberculosis and HIV services

The degree to which HIV and TB services are integrated at a clinic level is a function of joint planning and coordination of different clinic teams and systems. Uyei et al. (2016) developed and validated the Degree of Integrated Tuberculosis and HIV Service Delivery tool (Additional file 3), which quantifies the extent to which respondents perceived their clinic processes and systems to be organized and prepared for offering integrated HIV/TB services (Cronbach’s alpha of > 0.70) [28]. The tool measured eight sub-scales, namely, integrated TB and ART service delivery, availability of policies and protocols, integrated TB and pre-ART service delivery, same clinicians for both TB and HIV services, TB infection control, co-operation between TB and ART staff, TB screening, and clinician awareness of patient’s co-infection status. Sub-scale items are phrased as statements to which respondents could agree or disagree on a 5-point Likert-type scale; 1=Strongly Disagree and 5=Strongly Agree. The tool was administered at baseline and months 6, 12, and 18.

### HIV and TB process indicators

The parent study collected data on HIV-TB process indicators in both study arms from clinic registers and patient electronic database downloads. Monthly summary data on the number of patients that received a service (numerator) and number of patients who were eligible for a service (denominator) were collected and proportions calculated to monitor improvement for each HIV-TB process indicator.

### Focus group discussions with clinic staff

Clinic staff from both arms were recruited to participate in a study exit interview. The exit interviews were conducted as FGDs and designed to assess understanding of integrated HIV-TB service delivery, describe experiences of the QI clinic staff in implementing QI methods and document any improvement efforts of the SOC clinics. The FGDs were an opportunity to collect any insights on OCFs that were missed by the surveys. A purposive sample of clinic staff were recruited based on category of staff, availability and years spent in the clinic (at least 1 year). FGDs were conducted, using a semi-structured interview guide that was developed in-house (Additional file 4).

FGDs were conducted primarily in isiZulu and voice recorded. All participating clinic staff provided signed consent. Voice recordings were transcribed verbatim and then translated to English for analyses. Two study staff read the transcripts separately and extracted themes, including any barriers or facilitators to implementing QI or HIV-TB service integration. Themes were compared and common themes adopted. Direct quotes that supported a theme were highlighted.

Eleven FGDs involving 43 clinic staff were conducted. Six FGDs with an average of three participants each were from the QI arm and five FGDs with an average of four participants were from the SOC arm. In the QI arm, there were 16 female and four male participants and the mean number of years served in the clinic was 5.5 years (min-max: 1–15). In the SOC arm there were 18 female and three male participants and the mean number of years served in the clinic was 6.8 years (min-max:1–16).

### Recruitment of clinic staff

Participation in the surveys and FGDs were offered to professional nurses, enrolled nurses, lay counsellors, and data capturers. Written consent and at least 1 year of full-time employment were the minimum criteria. At baseline, we approached clinic staff in both the QI and SOC arms and gauged their interest for participation in the surveys once every 6 months. It was neither practical nor possible to administer the surveys to all clinic staff, hence, we recruited one team member from each staff category. During the study, we attempted to administer the survey to the same team member; however, work demands, time constraints, vacation leave, and absenteeism made this impossible. If the team member was not available, that individual was replaced with another team member from the same staff category in the same clinic. All surveys and FGDs were conducted in private spaces within the clinic.

### Data collection and management

Between 01 December 2016 to 1 June 2017, clinic infrastructure data were collected from all 40 study clinics. Surveys were paper-based and devoid of any identifiers that could link responses to a clinic staff member. All completed surveys were faxed to the study offices and electronically captured.

### Statistical analysis

The COACH survey and Degrees of Integrated TB and HIV services survey were used to develop a score for supportive contexts for change and the extent to which clinic teams were organized to offer integrated HIV and TB services, respectively. Both surveys were scored as follows : sub-scale means were calculated by adding up all responses and dividing by the number of items in that sub-scale. A total score was calculated by adding all sub-scale means and dividing by the number of sub-scales. A clinic’s score was calculated as the mean of all clinic staff who completed the survey. A cluster mean was calculated as the mean of clinic means in that cluster and finally, the study arm mean was the mean of all cluster means. The highest possible mean for both surveys was five. Means were converted to percentages by dividing by 5 and multiplying by 100. This was done to make survey scores standardised with other scores. If a survey question was missed by the researcher, a score for that question was replaced by the mean of all other items in that sub-scale.

Responses to items in the CPT were ‘Yes’ or ‘No’ responses and coded as a one or zero, respectively. As per Table 1, mean scores for physical infrastructure, staffing availability, flexibility of clinic hours, monitoring data for improvement and leadership support, were calculated for each clinic by adding all items in the sub-scale and dividing by the number of items in that sub-scale. The mean cluster score was the mean of all clinic scores in that cluster. The study arm score was the mean of the cluster score means.

A *t*-test was used to compare scores between the QI and SOC arms. We compared baseline and month 12 scores for the COACH and Degrees of Integrated TB and HIV services as the QI intervention was at its full strength during this period. Linear mixed modelling was conducted to determine which OCFs best predicted improvements for each HIV-TB process indicator. Each OCF were analysed separately in the model adjusted for study arm, time, and the interaction of study arm and time. The model assumed an exchangeable covariance and time was nested within the cluster for HIV-TB process indicators. The statistical software used was STATA, version 15.1.

### Ethics approval

The study was approved by the University of KwaZulu-Natal Biomedical Research Ethics Committee (BF 108/14). All clinic staff who agreed to complete a survey or who participated in FGDs, signed an informed consent form in English or *isiZulu.*

## Results

**Fig. 2:**
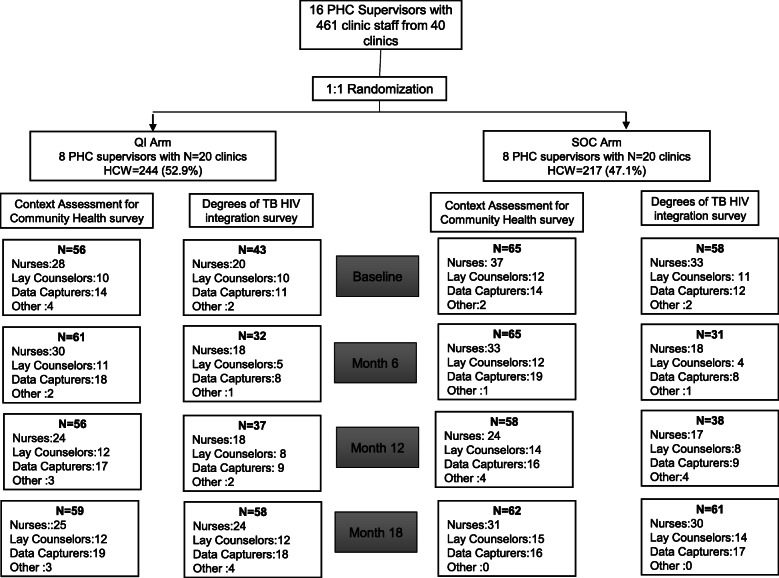
Clinic staff categories that responded to surveys at baseline and months 6, 12, and 18. QI, quality improvement; SOC, standard of care; PHC, primary health care

Across the 40 study clinics, a total of 461 clinic staff were available for this sub-study and 209 (45.3%) completed at least one survey (Fig. 2). Of the 209 clinic staff, 97 (46.4%) and 112 53.6%) were from the QI and SOC arms, respectively (Table 2). In the QI and SOC arm, 51.5% (50/97) and 54.5% (61/112) of respondents were nurses (Table 2). Most respondents (>80%) were female. The mean years of experience was 8.8 years [standard deviation (SD)=4.4] and 8.4 years (SD=5.4) in the QI and SOC arms, respectively.

**Table 2:** Characteristics of healthcare workers who participated in the study

Characteristics	QI arm	SOC arm	Total
	***n*** = 97	***n*** = 112	***N*** = 209
Mean age (years), mean (SD)	39.7 (9.4)	38.7 (8.9)	39.2 (9.1)
Female *n* (%)	81 (83.5)	97 (86.6)	178 (85.2)
Category of staff—*n* (%)			
Nurse categories			
Facility managers	12 (12.4)	9 (8.0)	21 (10.0)
Professional nurses	16 (16.5)	22 (19.6)	38 (18.2)
Enrolled nurses	22 (22.7)	30 (26.8)	52 (24.9)
Data capturers	22 (22.7)	22 (19.6)	44 (21.1)
Lay counsellors	17 (17.5)	25 (22.3)	42 (20.1)
Other	8 (8.2)	4 (3.6)	12 (5.7)
Mean years of experience, mean (SD) [min-max]	8.8 (4.4) [1–22]	8.4 (5.4) [1–34]	8.6 (4.9) [1–34]

*QI* quality improvement, *SD* standard deviation, *SOC* standard of care

A comparison between QI clinics and SOC clinics, showed similar access to basic services and staffing (Table 3). The QI arm had more high-volume clinics than the SOC group (14% versus (vs) 11 %). The mean monthly headcount in high-volume clinics were similar in both arms (Table 3).

**Table 3:** Clinic characteristics of the quality improvement arm and standard of care arm clinics

Clinic characteristic	Description	QI clinics (***N*** = 20)	SoC clinics (***N*** = 20)
Clusters per district (*n*)	KCD	5	4
	Ugu	3	4
Access to basic services one month prior to study enrolment *n* (%)	Electricity	18 (90)	19 (95)
	Water	16 (80)	17 (85)
	Telephone services	19 (95)	18 (90)
	Internet	2 (10)	0 (0)
Clinic operating hours *n* (%)	Normal working hours	5 (25)	4 (20)
	Extended working hours	15 (75)	16 (80)
High and low patient volume clinics^†^	Low volume clinics *n* (%)	6 (30)	9 (45)
	Low volume clinics mean (min–max)	1770 (1262–2383)	1755 (575–2380)
	High volume clinics, *n* (%)	14 (70)	11 (55)
	High volume clinics, mean (min–max)	4708 (2521–9638)	4029 (2577–6468)
Staff complement mean (min–max)	Low volume clinics		
	NIMART trained nurses	2 (1–3)	2 (2–3)
	TB trained nurses	2 (1–3)	2 (1–3)
	Enrolled nurses	1 (1–2)	1 (1–2)
	Data Capturers	1 (1)	2 (1–2)
	Lay counsellors	1 (1–2)	2 (1–2)
	Community caregivers	12 (5–18)	10 (4–32)
	High volume clinics		
	NIMART trained nurses	5 (1–11)	5 (2–12)
	TB trained nurses	2 (1–4)	3 (1–8)
	Enrolled nurses	2 (1–3)	2 (1–3)
	Data capturers	2 (1–3)	2 (1–3)
	Lay counsellors	3 (1–7)	2 (1–4)
	Community caregivers	16 (1–34)	18 (6–41)

Clustering was not considered for Table 2

^†^High volume clinics were defined as having a mean patient volume of > 2500 and low volume was defined as a patient volume ≤ 2500 per month

### Differences in Integrated HIV-TB service delivery performance

The parent study evaluated improvement in HIV-TB process indicators in the QI arm at baseline and post QI intervention (defined as months 13-18) [29]. Of the eight HIV-TB process indicators, we were unable to intervene on and analyze cotrimoxazole therapy and retention in care for HIV-TB patients, due to large amounts of missing data and limited study time and funds. An integrated patient electronic database was implemented in both study arms. Supplementary Figures 1 (A-F), shows the proportions achieved at baseline and post-QI intervention in the QI and SOC arms. In the QI group, IPT initiation rates improved by 60.5%, (Supplementary Figure 1D) [29]. In comparison the SOC arm improved by 23.1%. Modest improvements are noted in the QI and SOC for HIV testing services (9.7% versus 2.9%), HIV testing services in TB patients (7.6% versus 9.2%), TB screening (9.0% versus 7.7%) and viral load testing (10.8% versus 15.3%).

### Comparison of organizational contextual factors in QI and SOC arms

The mean scores achieved for OCFs measured in the QI and SOC arms are compared in Table 4. There were no OCF scores that were statistically significantly different between the QI and SOC arms. The largest difference in scores was observed in Physical Infrastructure which was 78.9% and 64.7% in the QI and SOC arms respectively; *p* = 0.058. The QI arm achieved a score of 46% for Leadership support visits versus 57.4% scored in the SOC arm; *p* = 0.265. The QI and SOC groups scored similarly in monitoring data for improvement (63.3% vs 65%; *p* = 0.875); however, both groups demonstrated a very wide range in scores, with some clinics scoring 100% in both groups.

**Table 4:** Comparison of organizational contextual factor (OCF) scores between QI and SOC groups

Organizational contextual factors	QI arm (*N* = 8)		SOC arm (*N* = 8)		*p*-value
	Mean (%)	Range (%)	Mean (%)	Range (%)	
Physical infrastructure	78.9	(66.7–90.5)	64.7	(42.9–80.0)	0.058
Key staff	95.8	(85.7–100)	92.0	(80.0–100)	0.270
Flexibility of clinic hours	66.9	(25–100)	65.5	(0–100)	0.900
Monitoring data for improvement (MDI)	63.3	(38.9–100)	65.0	(41.7–100)	0.875
Leadership support	46.0	(25.0–75.0)	57.4	(25.0–100)	0.265
Supportive context for change (baseline)^#^	77.5	(72.6–78.8)	79.0	(74.1–84.6)	0.248
Supportive context for change (month 12)^#^	76.2	(73.4–81.8)	79.7	(72.1–92.0)	0.128
Degree of integrated HIV-TB services (baseline)^#^	77.1	(72.8–82.9)	76.7	(66.7–82.4)	0.916
Degree of integrated HIV-TB services (month 12)^#^	74.1	(68.4–80.2)	80.1	(76.7–81.7)	0.916

*QI* quality improvement, *SOC* standard of care

^#^Mean scores were converted to percentages for comparability

The QI and SOC arms achieved scores of 77.5% and 79.0%, respectively at baseline, on the COACH survey (Table 4). After 12 months in the study, the QI and SOC arms scored 76.2% versus 79.7%, respectively; *p* = 0.128. After scoring the Degrees of integrated HIV-TB service delivery survey, the QI and SOC arm scored 77.1% and 76.1% respectively, at baseline. After 12 months in the study, QI and SOC arm, scored 74.1% and 80.1% respectively, *p* = 0.916.

### Organizational contextual factors associated with IPT initiation rates

While improvements were noted in HIV testing, TB screening and viral load monitoring, regression analyses were not possible in these indicators due to the small improvements made and the regression models did not converge. We used IPT initiation rates as the outcome variable in our regression analyses. Table 5 shows the bi-variate linear mixed modelling that tested for associations between each OCF and IPT initiation rates adjusted for time, study group and the interaction between study group and time. MDI was significantly associated with increasing IPT initiation rates (*β* = 0.04; *p* = 0.004). All other OCFs showed no statistically significant association with IPT initiation rates. In every bi-variate linear mixed model, the interaction of study group and time was significantly associated with increasing IPT initiation rates, suggesting that exposure to QI over time is predictive of increasing IPT performance irrespective of the influence of the OCF (*β* = 0.012; *p* = 0.004).

**Table 5:** Linear mixed models testing associations between organizational contextual factors and isoniazid preventive therapy

Organizational contextual factors	Coefficient (***β***)	Standard error (SE)	95% confidence interval (CI)		***p***-value
**Physical infrastructure**	0.002	0.003	− 0.005	0.008	0.605
Study group	− 0.006	0.094	− 0.190	0.178	0.950
Time (months)	0.008	0.003	0.002	0.014	**0.012**
Study group*Time	0.012	0.004	0.004	0.020	**0.004**
Constant	0.335	0.222	− 0.099	0.769	0.131
**Flexibility of clinic hours**	0.001	0.001	− 0.001	0.004	0.277
Study group	0.016	0.080	− 0.141	0.173	0.842
Time (months)	0.008	0.003	0.002	0.014	**0.012**
Study group*Time	0.012	0.004	0.004	0.020	**0.004**
Constant	0.357	0.099	0.163	0.551	< 0.001
**Monitoring data for improvement**	0.004	0.002	0.001	0.008	**0.004**
Study group	0.026	0.069	− 0.110	0.161	0.712
Time (months)	0.008	0.003	0.002	0.014	**0.012**
Study group*Time	0.012	0.004	0.004	0.020	**0.004**
Constant	0.156	0.112	− 0.063	0.374	0.163
**Leadership support**	0.003	0.002	0.000	0.006	0.056
Study group	0.053	0.078	− 0.099	0.205	0.494
Time (months)	0.008	0.003	0.002	0.014	**0.012**
Study group*Time	0.012	0.004	0.004	0.020	**0.004**
Constant	0.267	0.107	0.057	0.477	0.013
**Supportive context for change (month 12)**	− 0.009	0.008	− 0.024	0.007	0.267
Study group	− 0.012	0.084	− 0.178	0.153	0.884
Time (months)	0.008	0.003	0.002	0.014	**0.012**
Study group*Time	0.012	0.004	0.004	0.020	**0.004**
Constant	1.137	0.626	− 0.089	2.364	0.069
**Supportive context for change (month 12 adjusted for baseline)**	− 0.014	0.008	− 0.030	0.002	0.08
Study group	0.002	0.081	− 0.158	0.161	0.98
Baseline score	0.023	0.014	− 0.005	0.050	0.11
Time	0.008	0.003	0.002	0.014	**0.012**
Study group*Time	0.012	0.004	0.004	0.020	**0.004**
Constant	− 0.198	1.022	− 2.201	1.806	0.85
**Degree of integrated HIV-TB services (month 12)**	0.009	0.013	− 0.016	0.034	0.49
Study group	0.016	0.082	− 0.144	0.175	0.85
Time (months)	0.008	0.003	0.002	0.014	**0.012**
Study group*Time	0.012	0.004	0.004	0.020	**0.004**
Constant	0.019	0.614	− 1.185	1.223	0.98
**Degree of integrated HIV-TB services (adjusted for baseline)**	0.010	0.013	− 0.015	0.036	0.43
Study group	0.083	0.109	− 0.130	0.296	0.45
Baseline score	0.018	0.019	− 0.020	0.056	0.35
Time	0.008	0.003	0.002	0.014	**0.012**
Study group*Time	0.012	0.004	0.004	0.020	**0.004**
Constant	− 0.957	1.208	− 3.324	1.411	0.43
**District**	− 0.107	0.067	− 0.238	0.025	0.111
Study group	0.005	0.078	− 0.147	0.157	0.951
Time (months)	0.008	0.003	0.002	0.014	**0.012**
Study group*Time	0.012	0.004	0.004	0.020	**0.004**
Constant	0.499	0.065	0.372	0.625	< 0.001

Each model is adjusted for study group and time

### Clinic staff reflections on integrated HIV-TB service delivery and improvement activities

Barriers and facilitators to integrated HIV-TB service delivery extracted from the FGDs were related to (i) Understanding of what constitutes HIV-TB services, (ii) Awareness of gaps in HIV-TB service delivery (iii) Motivation to make improvements.

#### Understanding of integrated HIV-TB services

Understanding of integrated HIV-TB service delivery was similar in both study arms, with one exception, the mention of IPT to prevent TB. Focus group participants in both arms emphasized testing and screening for both diseases at the same clinic visit, linkage to TB and HIV treatment, and a single file system. Nurses in the QI clinics provided more comprehensive definitions of what it means to offer integrated HIV-TB services.

Coinfected patients should have one file for both TB/HIV. A person infected with HIV only should be screened for TB every visit. A person infected with TB only should be screened for HIV every 3 months. A person with both TB/HIV should be initiated to cotrimoxazole. Those that do not have TB but have HIV should be on INH to be prevented from contracting TB. (QI group, nurse)

#### Awareness of service delivery gaps

Lack of monitoring and evaluation of the IPT programme emerged as a possible reason for the low baseline IPT initiation rates in the QI clinics. Clinic staff in the QI arm reported being unaware that IPT initiation rates were low until it was highlighted during QI activities and the data was revealed to them. When asked to comment on how QI has improved HIV-TB integration, the QI group (without being prompted about IPT initiation) expressed how the QI highlighted IPT initiation and performance.

...things like IPT, IPT coverage, initiating IPT within 28 days of ART and all of that, you do not realize it is a problem until you start plotting and seeing what is happening. It also has helped to see staff performance (QI group, Professional nurse)

In the SOC clinics, three nurses reported receiving regular feedback from the District Health Offices and facility managers, on service delivery gaps.

#### Motivation to make improvements

In the QI clinics, interviewees mentioned several facilitators to making improvements in their clinic, including, a sense of shared responsibility for improvement efforts, clarity and transparency of individual roles and responsibilities, healthy competition, and benchmarking with other clinics in the collaborative. According to two nurses the QI trainings were too few learning sessions and limited to a small number of attendees which was a barrier to improvement in some clinics. Transfer of knowledge from learning session attendees to non-attendees was described as vague and incomplete which may have led to some clinic staff feeling ‘distanced’ from the QI intervention.

SOC clinics reported having access to resources for improvement, such as file audit templates, and access to expertise from local non-governmental organizations for data analysis, and development of performance charts. However, a lack of formal training and in-house experience in implementing improvement were barriers mentioned.

## Discussion

In the SUTHI trial, IPT initiation rates were dramatically improved in the QI arm compared to the SOC arm (Supplementary Figure 1D). After testing several OCFs for association with improvement in IPT initiation rates, we found that MDI and exposure to QI over time were the only factors significantly associated with increasing IPT initiation rates. Importantly, in this study MDI was a factor that was measured at baseline (before QI intervention implementation). In South Africa, an electronic health information system was designed for the purpose of collecting and analysing patient and process data and evaluating the HIV and TB programme for effectiveness. The practice of MDI is important for data-informed decision-making regarding the direction and effectiveness healthcare services and shows commitment to improving services to communities by clinic teams [30]. The range in MDI scores for the QI and SOC arms shows that all clinics were, to varying degrees, using routine data to monitor and improve the HIV-TB programme (Table 4). This suggests that the QI intervention was implemented in a context where the practice of using data for monitoring programme performance was already embedded and may have contributed to the success of the QI intervention in improving IPT initiation rates. ‘Monitoring services for action’ was a sub-scale of the COACH tool (Supplementary Table 1) and the high scores achieved by both study arms at baseline and even after month 12, supports this finding that the study setting had a culture of data use for improvement.

Although statistically significant, we acknowledge that the association between MDI and IPT initiation rates is very weak (low beta coefficient). IPT initiation rates improved by small increments every month. The small monthly differences in improvement and inclusion of several factors (study arm, time, interaction of study arm, and time) in the model produced low beta coefficients.

The importance of MDI is highlighted in other studies. Two systematic reviews that aimed to extract OCFs which predict outcomes in QI interventions, also identified the practice of MDI as key in influencing success of QI interventions [17, 31]. A South African-based study that adopted the Breakthrough Series Collaborative, reduced HIV transmission from mothers to infants from 7.6 to 5.0% in one sub-district [32]. The researchers partially attributed this success to an existing culture of using routine data to reflect on clinic performance which facilitated the adoption of QI and was familiar to front-line staff [32]. Access to good quality routine data that is relevant to front-line staff was a further driver of uptake of the intervention that led to a positive outcome [32].

The low IPT initiation rates at baseline suggest that this indicator was not being monitored or if it was, little was done to improve performance. The FGDs confirmed that the poor performance went undetected until the QI intervention began and IPT initiation rates were presented to clinics. QI interventions to improve IPT initiation rates have been successful in other countries. A national QI programme in Namibia improved IPT by 16 to 28% [33]. In comparison, a Nigerian study made a larger improvement in IPT (11% to 50%); however, their efforts were focused at one busy facility [34]. Both studies attribute this success to QI interventions building skills and confidence among clinic teams to make improvements.

In our study, the FGDs also confirm that QI clinics felt a positive shift in team motivation, in addition, there were other contextual factors that may have influenced the uptake of QI in the study. We observed that at baseline and at month 12, the QI and SOC arms achieved high scores on the degree of integrated HIV-TB services survey, which suggests that clinic teams are well coordinated and prepared to offer integrated services. The high ART initiation rates (> 90%) among co-infected patients (Supplementary Figure 1E) support this finding. The implementation of the integrated HIV and TB electronic data system is an indication of the commitment of the South African Department of Health to HIV-TB integration. Similarly, clinic teams in both study arms perceived high levels of supportiveness (high COACH scores) in their clinic to implement changes and this persisted at month 12 in the study. Given that QI clinics showed high levels of organization to offer integrated services and felt supported to make changes in their clinics, the QI intervention thrived in these clinics, particularly when poor performance was detected.

There were no significant differences in any OCF scores between QI and SOC arms. SOC arm clinics were similar to QI arm clinics for perceived organization to offer HIV-TB integrated services and supportiveness of contexts for change. The FGDs suggest that SOC clinics only lacked improvement “know how”. This is promising for any future scale-up of the QI intervention in this context which appears to have the correct conditions to embed a successful QI programme.

### Recommendations

Little is known of how best to foster the practice of MDI among clinic teams. Very few systematic reviews and intervention studies have been conducted on this topic [30]. Based on our findings and a small number of studies and systematic reviews that have been conducted, we recommend promoting the practice of MDI through making routine data accessible to clinic staff, ensuring good quality data, and improving the technical skills of clinic staff to use and generate reports from electronic health information systems. A Nigerian study tested the QI collaborative approach in enhancing prevention of mother-to-child services and included data quality as a key indicator for improvement [35]. As data quality improved, clinic teams reported increased levels of confidence in their clinic data and the use of improvement cycles using routine data [35]. Two studies demonstrated that electronic health information management systems are effective in assisting clinic teams and managers in making decisions about health programmes [36, 37]. Effectiveness studies of electronic information systems show that clinic teams will use data from electronic systems provided that the quality of data is accurate and reliable, reports are easily generated, skills and capacity to use the system is present, and no major hardware and software malfunctions occur [36, 37].

In addition, we found rapid assessments of organizational context using structured surveys useful to understanding the setting in which our QI intervention was implemented, and future QI initiatives should consider this approach and add to the knowledge base of how OCFs influence the success of QI.

### Limitations

The study has several limitations. The accuracy of data collected on surveys, such as the COACH survey, cannot be guaranteed. Social desirability bias may have influenced some responses particularly those of a sensitive nature, such as leadership and commitment to work. Two studies which tested the reliability of the COACH survey reported similar challenges of eliciting truthful responses and strongly recommend that confidentiality and privacy of data be emphasized to respondents [38, 39]. Despite assuring respondents’ confidentiality and anonymity, we received reports from study staff of hesitation among respondents to select answers that may reflect poorly on themselves, leaders, and the clinic team. Thus, COACH scores in this study may be inflated. In addition, we extended the use of the COACH survey to data capturers and lay counsellors, who may not have had some knowledge, such as clinic access to medication.

Using the validated measures repeatedly may not have been the ideal method to engage clinic staff. There were reports of “fatigue” among respondents regarding the time it takes to complete the surveys and being asked the same questions. The small sample size of 16 clusters restricted and affected the analyses. We were unable to perform regression models for each study arm. Secondly, the CPT was not a validated tool and the scoring system was developed by SG and CC. Future studies should consider development of a validated measure to assess aspects of physical infrastructure and resources in low- and middle-income countries. Thirdly, all OCF scores were at the cluster level and therefore highly summarized.

## Conclusion

This study has shown that QI interventions are successful in contexts where clinic teams are encouraged and supported to use routine data for improvement. IPT is an important intervention in interrupting the transmission of TB and is seldom prioritized for improvement. Capacitating clinic teams with QI skills and tools, fostering the practice of using routine data to monitor improvement, and removing any threats to using routine data may be the key to improving IPT initiations and other poorly performing indicators.

## Supplementary Information

**Additional file 1.**

**Additional file 2.**

**Additional file 3.**

**Additional file 4.**

**Additional file 5.**

**Additional file 6.**

## Data Availability

Individual participant data for completed studies is available on requests through the CAPRISA website; after approval of a proposal, data can be shared through a secure online platform.

## Abbreviations

ART: Antiretroviral Therapy
COACH: Context Assessment for Community Health
FGD: Focus Group Discussions
IHI: Institute for Healthcare Improvement
IPT: Isoniazid Preventive Therapy
MDI: Monitoring data for improvement
MES: Median Effect Size
OCF: Organizational Contextual Factors
PARIHS: Promoting Action on Research Implementation in Health Services
PHC: Primary Healthcare
QI: Quality Improvement
SD: Standard Deviation
SOC: Standard of Care
SUTHI: Scaling up TB HIV Integration
TB: Tuberculosis
TPT: TB Preventive Therapy
