# Supplementary material for: Organizational contextual factors that predict success of a quality improvement collaborative approach to enhance integrated HIV-tuberculosis services: a sub-study of the Scaling up TB/HIV Integration trial
Source: Implement Sci. 2021 Sep 17;16:88. doi: 10.1186/s13012-021-01155-7 (PMC8447673; doi:10.1186/s13012-021-01155-7)
Supplement: Supplementary file 1 — Additional file 1. [file 13012_2021_1155_MOESM1_ESM.pdf]

CAPRISA 013 Plate # 015

Visit Code 1 . __ __ __
Phase Month Interim

Participant ID
0 1 3 - __ __ - __ __ __ __
Study Site Participant

Visit Date __ __ __ __ __ __ __
dd MMM yy

Scaling up TB / HIV Integration (SUTHI)

Clinic Profile and Baseline Assessment Questionnaire - 1

A. Clinic Operating Hours :

**Mon - Fri :**	hr __ __ : min __ __	**To**	hr __ __ : min __ __
**Sat :**	hr __ __ : min __ __	**To**	hr __ __ : min __ __
**Sun :**	hr __ __ : min __ __	**To**	hr __ __ : min __ __
**Public Holidays :**	hr __ __ : min __ __	**To**	hr __ __ : min __ __

B. Infrastructure and Environment :

<u>Instruction for the interviewer</u> - As you probe for response to these questions, also look around the vicinity and fill in appropriate answers.

B1) Does the facility have any of the following?

			Yes / No	Yes / No	**Comments**
	Yes / No	How many?	They are been utilized	They are clean and organized	
1) Consultation rooms?	☐ ☐	__ __	☐ ☐	☐ ☐	
2) Designated patient waiting room	☐ ☐	__ __	☐ ☐	☐ ☐	
3) Private space vital signs check	☐ ☐	__ __	☐ ☐	☐ ☐	
4) Rooms for privacy during consultation	☐ ☐	__ __	☐ ☐	☐ ☐	
5) Private space for vital signs check	☐ ☐	__ __	☐ ☐	☐ ☐	
6) Pharmacy:	☐ ☐	__ __	☐ ☐	☐ ☐	
7) Cough Area ***Describe area***	☐ ☐	__ __	☐ ☐	☐ ☐	
8) Bathrooms	☐ ☐	__ __	☐ ☐	☐ ☐	
9) Staff kitchen	☐ ☐	__ __	☐ ☐	☐ ☐	
10) Toilets	☐ ☐	__ __	☐ ☐	☐ ☐	
11 Storage rooms	☐ ☐	__ __	☐ ☐	☐ ☐	
12) Other, specify : ____________	☐ ☐	__ __	☐ ☐	☐ ☐	

CAPRISA 013 Plate #016

Visit Code 1 . __ __ __
Phase Month Interim

Participant ID
0 1 3 - __ __ - __ __ __ __
Study Site Participant

Visit Date __ __ __ __ __ __ __
dd MMM yy

Scaling up TB / HIV Integration (SUTHI)

Clinic Profile and Baseline Assessment Questionnaire - 2

C. Communication and basic services availability :

	Yes / No		**Comments**
1) Is there always electricity in this clinic ?	☐ ☐		
2) Is there a back-up system in case of power failure ?	☐ ☐		
3) Is there an internet connection ?	☐ ☐		
4) Is there good water supply ?	☐ ☐		
5) Are there functional photocopying services in this clinic ?	☐ ☐	If ***NO,*** what then is being used ? ______________ ______________	
6) Is there a functional telephone service ?	☐ ☐	If ***NO,*** what then is being used ? ______________ ______________	

CAPRISA 013 Plate # 017

Visit Code 1 . ☐☐ ☐
Phase Month Interim

Participant ID
0 1 3 - ☐☐ - ☐☐☐☐
Study Site Participant

Visit Date ☐☐ ☐☐☐ ☐☐
dd MMM yy

Scaling up TB / HIV Integration (SUTHI)

Clinic Profile and Baseline Assessment Questionnaire - 3

D. Staffing :

D1. Does this clinic have any of the following ?

	Yes / No	If Yes, please state number	**Comments**
1) Medical officer	☐ ☐	☐☐	
2) Operational Manager	☐ ☐	☐☐	
3) PHC supervisors	☐ ☐	☐☐	
4) Registered nurses (RNS)	☐ ☐	☐☐	
4.1) RNS NIMART trained	☐ ☐	☐☐	
4.2) RNS initiated ARVS	☐ ☐	☐☐	
4.3) RNS TB trained	☐ ☐	☐☐	
5) Enrolled nurse	☐ ☐	☐☐	
5.1) ENS TB trained	☐ ☐	☐☐	
5.2) Enrolled nursing assistant	☐ ☐	☐☐	
6) Pharmacist	☐ ☐	☐☐	
7) Pharmacist assistant	☐ ☐	☐☐	
8) Data capturer	☐ ☐	☐☐	
9) Clinical support officer (CSO), Admin / Clerk	☐ ☐	☐☐	
10) Social worker	☐ ☐	☐☐	
11) Nutritional advisor	☐ ☐	☐☐	
12) Lay councilors	☐ ☐	☐☐	
13) Community care givers	☐ ☐	☐☐	
14) Cleaners	☐ ☐	☐☐	
15) Security	☐ ☐	☐☐	
Others, Specify :	☐ ☐	☐☐	
	☐ ☐	☐☐	

CAPRISA 013 Plate # 018

Visit Code 1 . [ ][ ] [ ]
Phase Month Interim

Participant ID

0 1 3 - [ ][ ] - [ ][ ][ ][ ]
Study Site Participant

Visit Date [ ][ ] [ ][ ][ ] [ ][ ]
dd MMM yy

Scaling up TB / HIV Integration (SUTHI)

Clinic Profile and Baseline Assessment Questionnaire - 4

D2. Are the routine duties rotated among nurses in this clinic ? Yes ☐ ☐ No

If ***YES*** to D2, complete questions below :	Weekly	Bi-Monthly	Monthly	Quarterly	**Comments if any**
1) How often are rotation among the nurses carried out ?	☐	☐	☐	☐	
2) How frequent does the PHC supervisor visit the clinic ?	☐	☐	☐	☐	
2.1) What does he/she do when they visit?					
3) How frequent does the district TB co-ordinator visit the clinic ?	☐	☐	☐	☐	
3.1) What does he/she do when they visit?					
4) How frequent does the district QA manager visit the clinic ?	☐	☐	☐	☐	
4.1) What does he/she do when they visit?					
5) How frequent does the district HAST co-ordinator visit the clinic ?	☐	☐	☐	☐	
5.1) What does he/she do when they visit?					
6) How frequent does the M&E team manager or M&E manager from the hospital visit the clinic ?	☐	☐	☐	☐	
6.1) What does he/she do when they visit?					
7) How frequent does the OM attend meetings arranged by the district DoH ?	☐	☐	☐	☐	

CAPRISA 013 Plate # 019

Visit Code 1 . _ _ _
Phase Month Interim

Participant ID
0 1 3 - _ _ - _ _ _ _
Study Site Participant

Visit Date _ _ _ _ _ _ _
dd MMM yy

Scaling up TB / HIV Integration (SUTHI)

Clinic Profile and Baseline Assessment Questionnaire - 5

E. Clinical stationery, infection control and equipment supplies :

E1. Are any of the following available ?

	Yes / No		How many?	Are they been utilized			**Comments**
				Yes	No	None at all	
1) Weighing scales (Adults& Paediatric)	☐	☐	☐☐	☐	☐	☐	
2) Blood pressure machines	☐	☐	☐☐	☐	☐	☐	
3 Stethoscope	☐	☐	☐☐	☐	☐	☐	
4) Thermometer	☐	☐	☐☐	☐	☐	☐	
5) Height measurement	☐	☐	☐☐	☐	☐	☐	
6) BMI Wheel	☐	☐	☐☐	☐	☐	☐	
7) Examination bed	☐	☐	☐☐	☐	☐	☐	
8) Wall mounted thermometer	☐	☐	☐☐	☐	☐	☐	
9) HIV test kits	☐	☐	☐☐	☐	☐	☐	
10) Stop watch	☐	☐	☐☐	☐	☐	☐	
11) Surgical masks	☐	☐	☐☐	☐	☐	☐	
12) N95 masks	☐	☐	☐☐	☐	☐	☐	
13) Vacutainer holders and needles	☐	☐	☐☐	☐	☐	☐	
14) Blood specimen bottles	☐	☐	☐☐	☐	☐	☐	
15) Sputum bottles	☐	☐	☐☐	☐	☐	☐	
16) Blood forms	☐	☐	☐☐	☐	☐	☐	
17) Sputum forms	☐	☐	☐☐	☐	☐	☐	
18) Clinical charts - Adults & Paeds	☐	☐	☐☐	☐	☐	☐	
19) Running water	☐	☐	☐☐	☐	☐	☐	
20) Liquid soap	☐	☐	☐☐	☐	☐	☐	

CAPRISA 013 Plate # 020

Visit Code 1 . [ ][ ] [ ]
Phase Month Interim

Participant ID

0 1 3 - [ ][ ] - [ ][ ][ ][ ]
Study Site Participant

Visit Date [ ][ ] [ ][ ][ ] [ ][ ]
dd MMM yy

Scaling up TB / HIV Integration (SUTHI)

Clinic Profile and Baseline Assessment Questionnaire - 6

E1 continued .....

	Yes / No		How many?	They are been utilized			**Comments**
				Yes	No	None at all	
21) Hand washing reminder posters	☐	☐	[ ][ ]	☐	☐	☐	
22) Disposable gloves	☐	☐	[ ][ ]	☐	☐	☐	
23) Colour coded waste bins	☐	☐	[ ][ ]	☐	☐	☐	
24) Bin liners	☐	☐	[ ][ ]	☐	☐	☐	
25) Disinfectants	☐	☐	[ ][ ]	☐	☐	☐	
26) Sharps container	☐	☐	[ ][ ]	☐	☐	☐	
27) Antiseptics	☐	☐	[ ][ ]	☐	☐	☐	
28) Disposable aprons	☐	☐	[ ][ ]	☐	☐	☐	

E2) How would you describe the information available in the clinic to patients from the charts, posters, pamphlets and leaflets on days of operation, basic health care prevention and management ?

Charts, posters, pamphlets and leaflets for basic prevention and management awareness of TB, HIV and TB-HIV co-infection at the clinic.	Please mark with a cross the appropriate box below<br>1 - Excellent<br>2 - Very good<br>3 - Good<br>4 - Fairly good<br>5 - Poor					Comments if any
	1	2	3	4	5	
1) Content	☐	☐	☐	☐	☐	
2) Pictorial illustrations	☐	☐	☐	☐	☐	
3) Correct Vocabulary, Grammar and Usage	☐	☐	☐	☐	☐	
4) Clarity	☐	☐	☐	☐	☐	
5) Translation in Zulu	☐	☐	☐	☐	☐	
6) Additional (Optional)	☐	☐	☐	☐	☐	

CAPRISA 013 Plate # 021

Visit Code 1 . Phase Month Interim

Participant ID

0 1 3 - [ ] - [ ]

Study Site Participant

Visit Date dd MMM yy

Scaling up TB / HIV Integration (SUTHI)

Clinic Profile and Baseline Assessment Questionnaire - 7

F. Pharmacy :

Items	Yes / No	Any of the drugs out of stock in the past 3 months? *(Please provide response with reasons)*	Comments if any
1) Temperature monitoring device available	☐ ☐		
2) Fridge available for temperature sensitive drugs ?	☐ ☐		
3) Are these ARVS drugs below available ?	☐ ☐		
3.1) Atroiza	☐ ☐		
3.2) Tenofovir	☐ ☐		
3.3) Lamivudine / Emtricitabine	☐ ☐		
3.4) Fixed dose combination	☐ ☐		
3.5) Zidovudine	☐ ☐		
3.6) Abacavir	☐ ☐		
3.7) Lopinavir / Ritonavir	☐ ☐		
3.8) Efavirapine	☐ ☐		
3.9) Nevirapine	☐ ☐		
4) Are these TB drugs below available ?	☐ ☐		
4.1) Rifafour	☐ ☐		
4.2) Rifinah	☐ ☐		
4.3) Streptomycin	☐ ☐		
5) Is Cotrimoxazole for adults and paeds available ?	☐ ☐		
6) Testing kits for HIV available ?	☐ ☐		
7) Any stock out of the testing kits in the past 3 months ?	☐ ☐	If Yes, comment of why - ____________	
8) Any expired ARV / TB drugs ?	☐ ☐	If Yes, how long ? - ____________	

CAPRISA 013 Plate # 022

Visit Code 1 . ☐☐ ☐
Phase Month Interim

Participant ID
0 1 3 - ☐☐ - ☐☐☐☐
Study Site Participant

Visit Date ☐☐ ☐☐☐ ☐☐
dd MMM yy

Scaling up TB / HIV Integration (SUTHI)

Clinic Profile and Baseline Assessment Questionnaire - 8

G. Guidelines and Protocols :

Which of the GUIDELINES listed among these item are available in this clinic ?	Available<br>Yes / No	If available, give the year available	Are the available documents been utilized ?<br>Yes / No	Comments
1) Consolidated HIV / PMTCT guidelines	☐ ☐	☐☐☐☐	☐ ☐	
2) Adult TB guidelines	☐ ☐	☐☐☐☐	☐ ☐	
3) Pediatric TB guidelines	☐ ☐	☐☐☐☐	☐ ☐	
4) MDR TB guidelines	☐ ☐	☐☐☐☐	☐ ☐	
5) Infection prevention and control guidelines	☐ ☐	☐☐☐☐	☐ ☐	
6) Clinical mentorship guidelines	☐ ☐	☐☐☐☐	☐ ☐	
7) Quality Improvement SOP	☐ ☐	☐☐☐☐	☐ ☐	
8) Data Management guidelines	☐ ☐	☐☐☐☐	☐ ☐	

H. Data Collection Tools and Statistics :

Data Collection Tools	Available<br>Yes / No	Utilized<br>Yes / No	Comments
1) Individual patient folders and sheets	☐ ☐	☐ ☐	
2) Registers :			
2.1) PHC Tick register	☐ ☐	☐ ☐	
2.2) IPT register	☐ ☐	☐ ☐	
2.3) HCT register	☐ ☐	☐ ☐	
2.4) TB suspect register	☐ ☐	☐ ☐	
2.5) TB treatment register	☐ ☐	☐ ☐	
2.6) Manual art register	☐ ☐	☐ ☐	
3) Tier.NET	☐ ☐	☐ ☐	
4) DHIS	☐ ☐	☐ ☐	

Staff Initials ☐☐☐ Date completed ☐☐ ☐☐☐ ☐☐

CAPRISA 013 Plate # 023

Visit Code 1 . ☐☐ ☐
Phase Month Interim

Participant ID
0 1 3 - ☐☐ - ☐☐☐☐
Study Site Participant

Visit Date ☐☐ ☐☐☐ ☐☐
dd MMM yy

Scaling up TB / HIV Integration (SUTHI)

Clinic Profile and Baseline Assessment Questionnaire - 9

H. Data Collection Tools and Statistics :

Data Collection Tools	Available Yes / No	Utilized Yes / No	Comments
5) Tallies and statistics used	☐ ☐	☐ ☐	
5.1) Daily tally book	☐ ☐	☐ ☐	
5.2) Weekly tally book	☐ ☐	☐ ☐	
5.3) Monthly summary	☐ ☐	☐ ☐	
5.4) Quarterly statistics	☐ ☐	☐ ☐	
6) Are TB and HIV files placed together-one filing system	☐ ☐	☐ ☐	
7) Is there an electronic single file system for HIV/TB co-infected clients ?	☐ ☐	☐ ☐	
8) Is the clinic signed off (live site able to produce monthly and quarterly reports) ?	☐ ☐	☐ ☐	
9) Does the clinic use TIER.net to obtain mortality data ***NB - Please probe for any other than TIER and document it (them)***	☐ ☐	☐ ☐	
10) Is there a system in place to ensure quality of data on TIER.net ?	☐ ☐	☐ ☐	
11) Does the clinic use data capture for anything ?	☐ ☐	☐ ☐	***If Yes, explain what for....*** ________________
12) Does the clinic hold information meetings (evidence) ?	☐ ☐	☐ ☐	

I. Clinical services :

**Are these TB / HIV services offered :**	Yes / No	Comments
1) TB / HIV education (evidence based)	☐ ☐	
2) ARV first line management	☐ ☐	
3) ARV second line management	☐ ☐	
4) DNA PCR	☐ ☐	
5) VCT	☐ ☐	
6) PICT	☐ ☐	

CAPRISA 013 Plate # 024

Visit Code 1 . __ __ __
Phase Month Interim

Participant ID
0 1 3 - __ __ - __ __ __ __
Study Site Participant

Visit Date __ __ __ __ __ __ __
dd MMM yy

Scaling up TB / HIV Integration (SUTHI)

Clinic Profile and Baseline Assessment Questionnaire - 10

**Are these TB / HIV services offered :**	Yes / No	Comments
7) TB SCREENING	☐ ☐	
8) Genexpert	☐ ☐	
9) Sputum microscopy	☐ ☐	
10) MDR TB treatment initiation	☐ ☐	
11) MDR TB treatment for clients initiated in MDR facilities	☐ ☐	
12) PMTCT	☐ ☐	
13) Tracing of TB contacts	☐ ☐	
14) Initiation of children into ARV	☐ ☐	

CLINIC SUPPORT :

Data Collection Tools	Available Yes / No	Utilized Yes / No	Comments
1) Is there an active clinic committee	☐ ☐	☐ ☐	
2) Is there any supporting organization in HIV and TB programs	☐ ☐	☐ ☐	***If Yes, name of organization*** ______
3) Is there any NGO / FBO / CBO that supports / assists in HIV and TB programs in this clinic ?	☐ ☐	☐ ☐	***If Yes, name of organization*** ______
4) Is there any NGO / FBO / CBO that supports / assists in HIV and TB programs in this community ?	☐ ☐	☐ ☐	***If Yes, name of organization*** ______
5) Is there support from the district office in HIV and TB programs ?	☐ ☐	☐ ☐	***If Yes, name of organization*** ______

CAPRISA 013 Plate # 025

Visit Code 1 . ☐☐ ☐
Phase Month Interim

Participant ID
0 1 3 - ☐☐ - ☐☐☐☐
Study Site Participant

Visit Date ☐☐ ☐☐☐ ☐☐
dd MMM yy

Scaling up TB / HIV Integration (SUTHI)

Clinic Profile and Baseline Assessment Questionnaire - 11

J. Existing Quality Improvement (QI) structure and initiatives in the clinic :

Questions :	Yes / No	Comments
1) Has there been any QI initiative in this clinic in the past 1 year ?	☐ ☐	***If Yes, provide brief details or list of the initiatives***<br>____________________<br>____________________
2) Is there a QI team in the clinic ? (If Yes, please list the designations of the team members - use comment section)	☐ ☐	
3) How frequently does the team meet ?<br>Weekly ☐ ☐ Bi-monthly<br>Monthly ☐ ☐ Quarterly<br>Other : ____________________		
4) What current ideas is been tested by the team ?<br>____________________<br>____________________		
5) Is there an external organization or unit supporting the QI work in your clinic ?	☐ ☐	***If Yes, provide name(s) and when.***<br>____________________<br>____________________
6) Has any of the staff been on QI ?	☐ ☐	***If Yes, mention role.***<br>____________________<br>____________________<br>____________________
