# Supplementary material for: Organizational contextual factors that predict success of a quality improvement collaborative approach to enhance integrated HIV-tuberculosis services: a sub-study of the Scaling up TB/HIV Integration trial
Source: Implement Sci. 2021 Sep 17;16:88. doi: 10.1186/s13012-021-01155-7 (PMC8447673; doi:10.1186/s13012-021-01155-7)
Supplement: Supplementary file 2 — Additional file 2. [file 13012_2021_1155_MOESM2_ESM.pdf]

CAPRISA 013 Plate # 012

Visit Code 1 . __ __ __
Phase Month Interim

Participant ID

0 1 3 - __ __ - __ __ __ __
Study Site Participant

Visit Date __ __ / __ __ __ / __ __
dd MMM yy

Scaling up TB / HIV Integration (SUTHI)

Context Assessment for Community Health (COACH) - 1

Name of Interviewer :

Interviewer code : __ __

Name of District :

Name of Sub - District :

Name of Facility :

Participant code : __ __

NB for the interviewer, kindly read to the participant : Thank you for agreeing to participate in this survey. We appreciate your cooperation in sharing your perceptions to enable us identify factors in your work place that might influence the way knowledge is used.

To what extent do you agree with the following ?
Please mark the appropriate box with an X

	Items	Strongly Agree	Agree	No Opinion	Disagree	Strongly Disagree	Don't Know	Refused to Answer
Resources	1. My unit has enough workers with the right training and skills to do everything that needs to be done.	☐	☐	☐	☐	☐	☐	☐
	2. My unit has enough workers with the right training and skill to do their job in the best possible way. .	☐	☐	☐	☐	☐	☐	☐
	3. My unit has enough space to provide healthcare services. .	☐	☐	☐	☐	☐	☐	☐
	4. My unit has access to the transport and fuel that are needed to provide healthcare services	☐	☐	☐	☐	☐	☐	☐
	5. My unit has access to the communication tools (eg : telephone or radios) that are needed to provide healthcare services.	☐	☐	☐	☐	☐	☐	☐
	6. My unit has enough medicine to provide healthcare services	☐	☐	☐	☐	☐	☐	☐
	7. This facility has enough functional equipment to provide healthcare services.	☐	☐	☐	☐	☐	☐	☐
	8. My unit has enough disposable medical equipment such as facemask, N95, glove, needle and syringes to provide HIV and TB medication and HIV tests kits.	☐	☐	☐	☐	☐	☐	☐

CAPRISA 013 Plate # 013

Visit Code 1 . [ ][ ] [ ]
Phase Month Interim

Participant ID
0 1 3 - [ ][ ] - [ ][ ][ ][ ]
Study Site Participant

Visit Date [ ][ ] [ ][ ][ ] [ ][ ]
dd MMM yy

Scaling up TB / HIV Integration (SUTHI)

Context Assessment for Community Health (COACH) - 2

NB for the interviewer, kindly read to the participant : Thank you for agreeing to participate in this survey. We appreciate your cooperation in sharing your perceptions to enable us identify factors in your work place that might influence the way knowledge is used.

To what extent do you agree with the following ?
Please mark the appropriate box with an X

	Items	Strongly Agree	Agree	No Opinion	Disagree	Strongly Disagree	Don't Know	Refused to answer
	9. If the workload increases, my unit can get additional resources such as personnel, medicine and equipment.	☐	☐	☐	☐	☐	☐	☐
Community Engagement	10. In my unit, we ask community members what they think about the healthcare service that we provide.	☐	☐	☐	☐	☐	☐	☐
	11. In my unit, we listen to what community members think about the healthcare services we provide.	☐	☐	☐	☐	☐	☐	☐
	12. In my unit, we have meetings with community members to discuss health matters.	☐	☐	☐	☐	☐	☐	☐
	13. In my unit, we encourage community members to contribute to improving the health of the community facility.	☐	☐	☐	☐	☐	☐	☐
	14. In my unit, we encourage other organizations to contribute to the improving the health of the community.	☐	☐	☐	☐	☐	☐	☐
Monitoring services for action	15. I receive regular updates about my unit's performance based on information / data collected from this facility.	☐	☐	☐	☐	☐	☐	☐
	16. My unit discusses information / data from the facility in a regular, formal way, such as in regular schedule meetings.	☐	☐	☐	☐	☐	☐	☐
	17. My unit regularly uses its unit information / data to make plans for improving its healthcare services.	☐	☐	☐	☐	☐	☐	☐
	18. My unit regularly monitors its work by comparing it with the unit's action plans.	☐	☐	☐	☐	☐	☐	☐
	19. My unit regularly compares its work with national or other guidelines.	☐	☐	☐	☐	☐	☐	☐
	20. I have access to clinical practice guidelines.	☐	☐	☐	☐	☐	☐	☐
	21. I have access to other printed material for work (eg. textbooks, journals)	☐	☐	☐	☐	☐	☐	☐
	22. I have access to in-service training / workshop / courses.	☐	☐	☐	☐	☐	☐	☐

CAPRISA 013 Plate # 014

Visit Code 1 . ☐☐ ☐
Phase Month Interim

Participant ID
0 1 3 - ☐☐ - ☐☐☐☐
Study Site Participant

Visit Date ☐☐ ☐☐☐ ☐☐
dd MMM yy

Scaling up TB / HIV Integration (SUTHI)

Context Assessment for Community Health (COACH) - 3

To what extent do you agree with the following ? *Please mark the appropriate box with an X*		Rating Scale						
	Items	Strongly Agree	Agree	No Opinion	Disagree	Strongly Disagree	Don't Know	Refused to answer
Sources of Knowledge								
	24. I have access to the Internet.	☐	☐	☐	☐	☐	☐	☐
	25. I have access to electronic decision support (eg. mobile phone applications or other electronic devices to assist with care and decision making)	☐	☐	☐	☐	☐	☐	☐
Commitment to work	26. I am proud to work in this facility.	☐	☐	☐	☐	☐	☐	☐
Work culture	27. My unit is willing to use new healthcare practices such as guidelines and recommendations.	☐	☐	☐	☐	☐	☐	☐
	28. My unit helps me to improve and develop my skills	☐	☐	☐	☐	☐	☐	☐
	29. I am encouraged to seek new information on healthcare practices.	☐	☐	☐	☐	☐	☐	☐
	30. My unit works for the good of the patients and puts their needs first.	☐	☐	☐	☐	☐	☐	☐
	31. Members of the unit approach patients with respect.	☐	☐	☐	☐	☐	☐	☐
Leadership	32. I trust the unit leader	☐	☐	☐	☐	☐	☐	☐
	33. The leader handles stressful situations calmly.	☐	☐	☐	☐	☐	☐	☐
	34. The leader actively listens, acknowledges, and then responds to requests and concerns.	☐	☐	☐	☐	☐	☐	☐
	35. The leader effectively resolves any conflicts that arise.	☐	☐	☐	☐	☐	☐	☐
	36. The leader encourages the introduction of new ideas and practices.	☐	☐	☐	☐	☐	☐	☐
	37. The leader makes things happen.	☐	☐	☐	☐	☐	☐	☐
Correction of malpractices	38. Efforts are made to address any illegal practices by the care workers (moonlighting, taking money from patients)	☐	☐	☐	☐	☐	☐	☐

☐☐☐ Staff Initials

☐☐ ☐☐☐ ☐☐ Date completed
