# Supplementary material for: Organizational contextual factors that predict success of a quality improvement collaborative approach to enhance integrated HIV-tuberculosis services: a sub-study of the Scaling up TB/HIV Integration trial
Source: Implement Sci. 2021 Sep 17;16:88. doi: 10.1186/s13012-021-01155-7 (PMC8447673; doi:10.1186/s13012-021-01155-7)
Supplement: Supplementary file 3 — Additional file 3. [file 13012_2021_1155_MOESM3_ESM.pdf]

CAPRISA 013 Plate # 050

Page number 2

Participant ID

0 1 3 - __ __ - __ __ __ __

Study Site Participant

Visit Date __ __ / __ __ __ / __ __

dd MMM yy

Scaling up TB / HIV Integration (SUTHI)

Service Integration Survey - 1

Name of Interviewer :

Name of District :

Name of Sub - District :

Name of Facility :

Participant code : __ __

For the interviewer - Please read the statements to the participant and let them say how they agree or disagree.

Section 1 : Integration measures :

	Strongly Agree	Agree	No Opinion	Disagree	Strongly disagree	Don't Know	Refused to answer
1.1 At this clinic/CHC, TB and ARV patient records are always kept together in one folder.	☐	☐	☐	☐	☐	☐	☐
1.2 The patient folders provided to clinicians (i.e.,doctors and nurses) always indicates a patient's TB/HIV co-infection status.	☐	☐	☐	☐	☐	☐	☐
1.3 The system for scheduling appointments allows patients to schedule TB and pre-ARV consultations on the same day.	☐	☐	☐	☐	☐	☐	☐
1.4 The system for scheduling appointments allows patients to schedule TB and ARV consultations on the same day.	☐	☐	☐	☐	☐	☐	☐
1.5 All TB, pre-ARV, and/or ARV clinical staff are provided training on how to manage TB/HIV co-infected patients. Training can be provided by the clinic or an outside source (e.g., NGO or national training)	☐	☐	☐	☐	☐	☐	☐
1.6 The clinic space is ideally configured for effective TB infection control. *(For the Interviewer -Examples of TB infection control measures include proper air* circulation, providing coughing patients with masks, and safe sputum collection.)	☐	☐	☐	☐	☐	☐	☐
1.7 I think the organizational components of TB and ARV services at this clinic are well integrated. **NB FOR THE INTERVIEWER :** *Organizational components refers to patient's records, location of TB and HIV clinics...whether its separate or in one* space.	☐	☐	☐	☐	☐	☐	☐

CAPRISA 013 Plate # 051

Page number 2 | | |

Participant ID

0 1 3 - _ _ - _ _ _ _

Study Site Participant

Visit Date _ _ / _ _ _ / _ _

dd MMM yy

Scaling up TB / HIV Integration (SUTHI)

Service Integration Survey - 2

Section 2 : Clinical Integration - Structure :

	Strongly Agree	Agree	No Opinion	Disagree	Strongly disagree	Don't Know	Refused to answer
2.1 I am aware of a written guideline that stipulates that all TB patients must be tested for HIV.	☐	☐	☐	☐	☐	☐	☐
2.2 I am aware of a written policy for screening all HIV positive patients for TB.	☐	☐	☐	☐	☐	☐	☐
2.3 I am aware of a written protocol for referring ARV eligible patients to an ARV site.	☐	☐	☐	☐	☐	☐	☐
2.4a I am aware of written guidelines on how to manage patients who are co-infected with TB and HIV.	☐	☐	☐	☐	☐	☐	☐
2.4b I was provided a copy of the South African National Treatment guidelines.	☐	☐	☐	☐	☐	☐	☐
2.5a I am aware of a written protocol at this clinic/CHC that promotes coordinated or collaborative TB and ARV services.	☐	☐	☐	☐	☐	☐	☐
2.5b I was provided a copy of these guidelines.	☐	☐	☐	☐	☐	☐	☐
2.6 I am aware of a written TB infection control plan for this clinic that aims to reduce the spread of TB to patients and staff.	☐	☐	☐	☐	☐	☐	☐

Section 3 : Clinical Integration - Process : (TB and pre-ARV services)

	Strongly Agree	Agree	No Opinion	Disagree	Strongly disagree	Don't Know	Refused to answer
3.1 At this clinic/CHC, both TB and pre-ARV services are available	☐	☐	☐	☐	☐	☐	☐
3.2 All TB suspects are always offered on-site HIV testing.	☐	☐	☐	☐	☐	☐	☐
3.3 At this clinic/CHC, all known HIV-positive persons are always screened for TB at every clinic visit.	☐	☐	☐	☐	☐	☐	☐
3.4 A co-infected person can always receive TB and pre-ARV services in one visit.	☐	☐	☐	☐	☐	☐	☐
3.5 TB/HIV co-infected patients always go to two separate doctors: One for their TB and one for their HIV services.	☐	☐	☐	☐	☐	☐	☐
3.6 TB/HIV co-infected patients always go to two separate nurses: One for their TB and one for their HIV services.	☐	☐	☐	☐	☐	☐	☐

Participant ID

0 1 3 - __ - ____

Study Site Participant

Visit Date dd MMM yy

Scaling up TB / HIV Integration (SUTHI)

Service Integration Survey - 3

Clinical Integration - Process : (TB and ARV services)

	Strongly Agree	Agree	No Opinion	Disagree	Strongly disagree	Don't Know	Refused to answer
3.7 At this clinic/CHC, both TB and ARV services are always available.	☐	☐	☐	☐	☐	☐	☐
3.8 A patient can always receive TB and ARV services in one visit at this clinic.	☐	☐	☐	☐	☐	☐	☐
3.9 Co-infected patients always go to two separate doctors : One for their TB and one for their ARVs.	☐	☐	☐	☐	☐	☐	☐
3.10 TB/HIV co-infected patients on TB treatment and ARVs always go to two separate nurses : One for their TB and one for their HIV services.	☐	☐	☐	☐	☐	☐	☐
3.11 Every single HIV-positive patient on ARVs at this clinic is routinely screened for TB.	☐	☐	☐	☐	☐	☐	☐
3.12 Co-infected patients follow two separate adherence and support protocols : One for TB treatment adherence and one for ARV adherence.	☐	☐	☐	☐	☐	☐	☐

Clinical Integration - Process / General

	Strongly Agree	Agree	No Opinion	Disagree	Strongly disagree	Don't Know	Refused to answer
3.13 For each and every one of my patients, I always know if s/he is co-infected with TB and HIV.	☐	☐	☐	☐	☐	☐	☐
3.14 For each and every one of my patients, I always know of s/he is taking ARVs.	☐	☐	☐	☐	☐	☐	☐
3.15 For each and every one of my patients, I always know of s/he is on TB treatment.	☐	☐	☐	☐	☐	☐	☐
3.16 This clinic fully implements TB infection control practices.	☐	☐	☐	☐	☐	☐	☐

CAPRISA 013 Plate # 053

Page number 2

Participant ID
0 1 3 - __ __ - __ __ __ __
Study Site Participant

Visit Date __ __ / __ __ __ / __ __
dd MMM yy

Scaling up TB / HIV Integration (SUTHI)
Service Integration Survey - 4

Section 4 : Clinical Integration - Culture :

	Strongly Agree	Agree	No Opinion	Disagree	Strongly disagree	Don't Know	Refused to answer
4.1 I see my role as part of a joint TB/HIV effort and not just providing HIV or TB services only.	☐	☐	☐	☐	☐	☐	☐
4.2 The TB epidemic cannot be controlled without integrating HIV and TB services.	☐	☐	☐	☐	☐	☐	☐
4.3 If trained, I am willing to provide both TB and ARV services to patients.	☐	☐	☐	☐	☐	☐	☐
4.4 I think patients should follow different TB and ARV adherence and support guidelines : One for TB and one for ARVs	☐	☐	☐	☐	☐	☐	☐
4.5 Cooperation between TB and pre-ARV HIV clinical staff is highly encouraged at this clinic/CHC	☐	☐	☐	☐	☐	☐	☐
4.6 Cooperation between TB and ARV clinical staff is highly encouraged at this clinic/CHC.	☐	☐	☐	☐	☐	☐	☐
