# Supplementary material for: Organizational contextual factors that predict success of a quality improvement collaborative approach to enhance integrated HIV-tuberculosis services: a sub-study of the Scaling up TB/HIV Integration trial
Source: Implement Sci. 2021 Sep 17;16:88. doi: 10.1186/s13012-021-01155-7 (PMC8447673; doi:10.1186/s13012-021-01155-7)
Supplement: Supplementary file 4 — Additional file 4. [file 13012_2021_1155_MOESM4_ESM.pdf]

Focus group questions

- **RM to introduce the focus group members to CAPRISA staff present**
- **Everyone needs to say what their designation is at the clinic & How long they worked at the clinic**
- **Assure staff that their information will be confidential. Give them IC to sign (English or ZULU)**
- **Introduce purpose of focus group interviews**

1. When did you first hear about quality Improvement? From Whom?
    - What does quality improvement mean to you?
    - **For control clinics** Do you think this clinic is implementing quality improvement?
    - If yes, when did they start implementing? Is the clinic getting support or advice to implement QI? From whom? What have you learned about Quality Improvement /QI skills ?
    - Do you think the training you received on Quality Improvement was adequate ?
2. What is your understanding of TB/HIV service integration?
    - Can you list the essential services that make up integrated TB/HIV service delivery ?
    - **For intervention clinics:** Do you believe that the QI, will indeed improve TB/HIV service integration?
    - **For control clinics:** Have you and your team at the clinic undertaken any initiatives to ensure that there is integration of TB/HIV services ? What have you done ? Who started the initiative ?
3. **For control clinics: W**hat strategies does the clinic use to integrate TB and HIV services?
    - Patient flow
    - Staff training
4. Do you think that TB/HIV service integration can be improved in this clinic? What prevents the clinic from integrating these services to the standards you would like to see? Explore: staff knowledge, resources, human resource, infrastructure.
5. Do you think TB/HIV coinfected patients are managed effectively in this clinic?
6. **For control clinics :** IF QI not mentioned, has your TB/HIV service delivery got worse / better after implementing QI? How has this affected daily routine activities? (work culture, stock, waiting times, patient staff relations)
7. **For control clinics :** Did you receive support from management to deliver effective TB/HIV management?
    If yes, from whom? What kind of support were you given?
    If no support from management, would you like to be supported? What kind of support do you need?
8. **For intervention clinics :** Do you need more training on QI?
    Would you encourage other clinics to do QI?
    There is anything you think we should have talked about in this discussion and we did not mention?
9. Is there any element that you would add to the QI that you felt was missing?
10. Would you recommend that more clinics be initiated to the QI (why)?
11. Do you believe that the support you received from the QI is sufficient enough to last you even after the intervention has been completed?

12. Do you have intentions to follow all training received throughout the QI intervention period?
