# Supplementary material for: Organizational contextual factors that predict success of a quality improvement collaborative approach to enhance integrated HIV-tuberculosis services: a sub-study of the Scaling up TB/HIV Integration trial
Source: Implement Sci. 2021 Sep 17;16:88. doi: 10.1186/s13012-021-01155-7 (PMC8447673; doi:10.1186/s13012-021-01155-7)
Supplement: Supplementary file 5 — Additional file 5. [file 13012_2021_1155_MOESM5_ESM.pptx]

## Slide 1
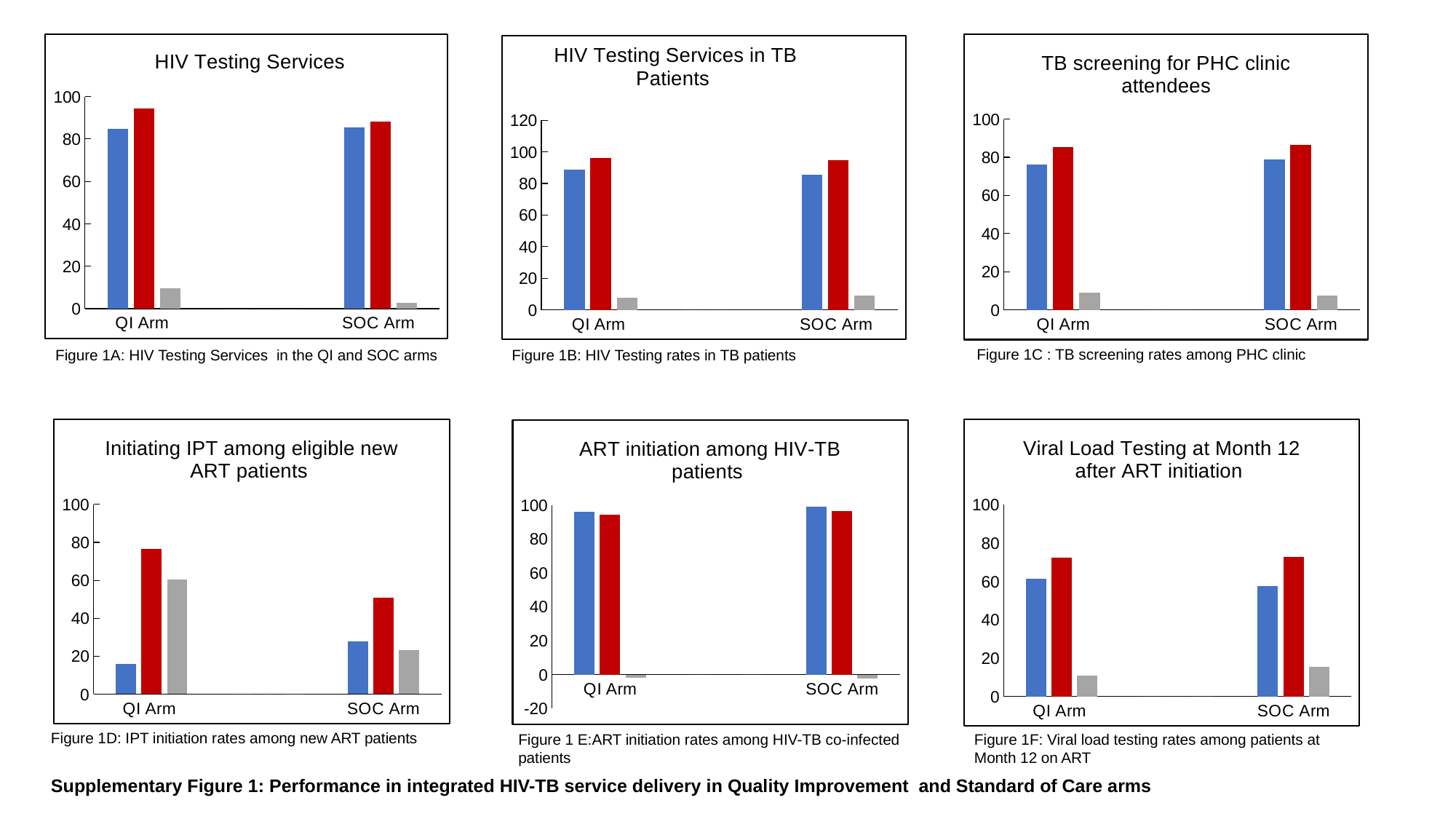

### Chart: TB screening for PHC clinic attendees
| Category | Baseline | Post QI | Improvement |
|---|---|---|---|
| QI Arm | 76.2 | 85.2 | 9.0 |
| | 0.0 | 0.0 | None |
| SOC Arm | 78.9 | 86.6 | 7.699999999999989 |
### Chart: HIV Testing Services
| Category | Baseline | Post QI | Improvement |
|---|---|---|---|
| QI Arm | 84.8 | 94.5 | 9.700000000000003 |
| | 0.0 | 0.0 | 0.0 |
| SOC Arm | 85.3 | 88.2 | 2.9000000000000057 |
### Chart: HIV Testing Services in TB Patients
| Category | Baseline | Post QI | Improvement |
|---|---|---|---|
| QI Arm | 88.7 | 96.3 | 7.599999999999994 |
| | 0.0 | 0.0 | None |
| SOC Arm | 85.7 | 94.9 | 9.200000000000003 |Figure 1C : TB screening rates among PHC clinic
Figure 1A: HIV Testing Services in the QI and SOC arms
Figure 1B: HIV Testing rates in TB patients
### Chart: Initiating IPT among eligible new ART patients
| Category | Baseline | Post QI | Improvement |
|---|---|---|---|
| QI Arm | 15.9 | 76.4 | 60.50000000000001 |
| | 0.0 | 0.0 | 0.0 |
| SOC Arm | 27.7 | 50.8 | 23.099999999999998 |
### Chart: Viral Load Testing at Month 12 after ART initiation
| Category | Baseline | Post QI | Improvement |
|---|---|---|---|
| QI Arm | 61.4 | 72.2 | 10.800000000000004 |
| | 0.0 | 0.0 | 0.0 |
| SOC Arm | 57.5 | 72.8 | 15.299999999999997 |
### Chart: ART initiation among HIV-TB patients
| Category | Baseline | Post QI | Improvement |
|---|---|---|---|
| QI Arm | 95.8 | 94.1 | -1.7000000000000028 |
| | 0.0 | 0.0 | 0.0 |
| SOC Arm | 98.9 | 96.5 | -2.4000000000000057 |Figure 1D: IPT initiation rates among new ART patients
Figure 1 E:ART initiation rates among HIV-TB co-infected patients
Figure 1F: Viral load testing rates among patients at Month 12 on ART
Supplementary Figure 1: Performance in integrated HIV-TB service delivery in Quality Improvement and Standard of Care arms
