# Supplementary material for: Organizational contextual factors that predict success of a quality improvement collaborative approach to enhance integrated HIV-tuberculosis services: a sub-study of the Scaling up TB/HIV Integration trial
Source: Implement Sci. 2021 Sep 17;16:88. doi: 10.1186/s13012-021-01155-7 (PMC8447673; doi:10.1186/s13012-021-01155-7)
Supplement: Supplementary file 6 — Additional file 6. [file 13012_2021_1155_MOESM6_ESM.docx]

| **COACH Subscales**  **Supplementary Table 1 : Mean scores achieved on sub-scales of the COACH tool in the QI and SOC Arms** | **Baseline** | | | **Month 6** | | | **Month 12** | | | | **Month 18** | | |
| --- | --- | --- | --- | --- | --- | --- | --- | --- | --- | --- | --- | --- | --- |
|  | n | Mean* | (SE) | n | Mean* | (SE) | n | | Mean* | (SE) | n | Mean* | (SE) |
| **Resources** |  |  |  |  |  |  |  | |  |  |  |  |  |
| QI arm | 56 | 3.27 | 0.10 | 61 | 3.34 | 0.10 | 56 | | 3.38 | 0.10 | 59 | 3.21 | 0.10 |
| SOC arm | 65 | 3.48 | 0.10 | 65 | 3.23 | 0.09 | 58 | | 3.11 | 0.10 | 62 | 3.05 | 0.10 |
| **Community Engagement** |  |  |  |  |  |  |  | |  |  |  |  |  |
| QI arm | 56 | 4.10 | 0.08 | 61 | 4.05 | 0.07 | 56 | | 3.93 | 0.08 | 59 | 3.96 | 0.08 |
| SOC arm | 65 | 4.11 | 0.07 | 65 | 4.03 | 0.07 | 58 | | 3.99 | 0.08 | 62 | 3.98 | 0.07 |
| **Monitoring services for action** |  |  |  |  |  |  |  | |  |  |  |  |  |
| QI arm | 56 | 4.10 | 0.08 | 61 | 4.11 | 0.08 | 56 | | 4.11 | 0.08 | 59 | 4.11 | 0.08 |
| SOC arm | 65 | 4.18 | 0.08 | 65 | 4.15 | 0.08 | 58 | | 4.16 | 0.08 | 62 | 4.17 | 0.08 |
| **Sources of knowledge** |  |  |  |  |  |  |  | |  |  |  |  |  |
| QI arm | 56 | 3.41 | 0.07 | 61 | 3.37 | 0.07 | 56 | | 3.28 | 0.07 | 59 | 3.38 | 0.07 |
| SOC arm | 65 | 3.43 | 0.07 | 65 | 3.37 | 0.07 | 58 | | 3.33 | 0.07 | 62 | 3.37 | 0.07 |
| **Commitment to work** |  |  |  |  |  |  |  | |  |  |  |  |  |
| QI arm | 56 | 3.88 | 0.15 | 61 | 4.05 | 0.14 | 56 | | 4.05 | 0.14 | 59 | 3.87 | 0.14 |
| SOC arm | 65 | 3.86 | 0.14 | 65 | 3.92 | 0.14 | 58 | | 3.89 | 0.14 | 62 | 3.59 | 0.14 |
| **Work culture** |  |  |  |  |  |  |  | |  |  |  |  |  |
| QI arm | 56 | 4.11 | 0.07 | 61 | 4.21 | 0.07 | 56 | | 4.09 | 0.07 | 59 | 4.03 | 0.07 |
| SOC arm | 65 | 4.14 | 0.07 | 65 | 4.14 | 0.07 | 58 | | 4.06 | 0.07 | 62 | 4.04 | 0.07 |
| **Leadership** |  |  |  |  |  |  |  | |  |  |  |  |  |
| QI arm | 56 | 4.15 | 0.10 | 61 | 4.15 | 0.10 | 56 | | 3.97 | 0.10 | 59 | 4.03 | 0.10 |
| SOC arm | 65 | 4.15 | 0.09 | 65 | 4.13 | 0.09 | 58 | | 4.09 | 0.10 | 62 | 3.95 | 0.10 |
| **Correction of malpractice** |  |  |  |  |  |  |  |  | |  |  |  |  |
| QI arm | 57 | 4.06 | 0.12 | 61 | 3.90 | 0.12 | 56 | 3.59 | | 0.12 | 60 | 3.82 | 0.12 |
| SOC arm | 65 | 4.13 | 0.11 | 65 | 3.92 | 0.11 | 58 | 3.85 | | 0.12 | 62 | 3.89 | 0.12 |
| **Overall COACH Score** |  |  |  |  |  |  |  |  | |  |  |  |  |
| QI arm | 56 | 3.89 | 0.06 | 61 | 3.90 | 0.06 | 56 | 3.80 | | 0.06 | 59 | 3.81 | 0.06 |
| SOC arm | 65 | 3.93 | 0.06 | 65 | 3.86 | 0.06 | 58 | 3.81 | | 0.06 | 62 | 3.75 | 0.06 |

COACH, Context Assessment for Community Health; QI, Quality Improvement; SE, Standard Error

*The highest possible mean score was 5 and scores greater than 3 were considered high scores
